# Recent advances in understanding of various chronic pain mechanisms through lysophosphatidic acid (LPA) receptor signaling

**DOI:** 10.1186/ar3561

**Published:** 2012-02-09

**Authors:** Hiroshi Ueda

**Affiliations:** 1 Division of Molecular Pharmacology and Neuroscience, Nagasaki University Graduate School Biomedical Sciences, Nagasaki 852-8521, Japan

## 

Lysophosphatidic acid (LPA) receptor (LPA1) signaling plays the key role in initiation of nerve injury-induced neuropathic pain [1-4]. LPA, which is produced in the spinal cord following the sciatic nerve injury causes a calpain-mediated demyelination of dorsal root fibers and sprouting through LPA1 receptor, leading to an induction of synaptic reorganization underlying allodynia. The LPA1 signaling also initiates the up-regulation of Ca_v_α_2_δ_1 _in DRG, leading to an enhancement of spinal pain transmission underlying hyperalgesia. Similar LPA1-mediated chronic abnormal pain and underlying mechanisms are observed in mouse models with Meth-A sarcoma surrounding sciatic nerve (cancer model) or with chemotherapy (paclitaxel). Central neuropathic pain following spinal nerve injury is now recently found to include the LPA1-mediated mechanisms. In contrast, (arthritic) inflammatory pain following Complete Freund Adjuvant treatment fails to show the involvement of LPA1 signaling. Thus it seems that many models of neuropathic pain, but not inflammatory pain model include LPA1-mediated mechanisms.

Recent studies revealed that another subtype LPA3 receptor plays a crucial role in neuropathic pain mechanisms in terms of LPA biosynthesis. Nerve injury and intrathecal administration of LPA increased the levels of lysophosphatidylcholine (LPC) and LPA in the spinal dorsal horn and dorsal root with peaks at 1 - 2 h. We obtained the evidence for in vitro LPA biosynthesis in spinal dorsal horn and dorsal root as well as in vivo one. In these studies we successfully identified the species of LPC and LPA molecules by use of Mass Spectrometery. Major species are the molecules with lipid chain 16:0, 18:0 or 18:1, and their contents were all time-dependently increased by nerve injury. Interestingly, there was an LPA-induced amplification of LPA biosynthesis through an activation of LPA3 receptor and microglia. The microglial involvement was found to play key roles as an initiation of neuropathic pain mechanisms including LPA3-mediated amplification of LPA biosynthesis.
